# First-Line Anti-Tubercular Drug Resistance of *Mycobacterium tuberculosis* in IRAN: A Systematic Review

**DOI:** 10.3389/fmicb.2016.01139

**Published:** 2016-07-28

**Authors:** Babak Pourakbari, Setareh Mamishi, Mona Mohammadzadeh, Shima Mahmoudi

**Affiliations:** ^1^Pediatric Infectious Disease Research Center, Tehran University of Medical SciencesTehran, Iran; ^2^Department of Infectious Diseases, Pediatrics Center of Excellence, Children's Medical Center, Tehran University of Medical SciencesTehran, Iran

**Keywords:** tuberculosis (TB), multidrug resistance tuberculosis (MDR), Iran

## Abstract

**Background:** The spread of drug-resistant tuberculosis (TB) is one of the major public health problems through the world. Surveillance of anti-TB drug resistance is essential for monitoring of TB control strategies. The occurrence of drug resistance, particularly multi-drug resistance *Mycobacterium tuberculosis* (MDR), defined as resistance to at least rifampicin (RIF) and isoniazid (INH), has become a significant public health dilemma. The status of drug-resistance TB in Iran, one of the eastern Mediterranean countries locating between Azerbaijan and Armenia and high-TB burden countries (such as Afghanistan and Pakistan) has been reported inconsistently. Therefore, the aim of this study was to summarize reports of first-line anti-tubercular drug resistance in *M. tuberculosis* in Iran.

**Material and Methods:** We systematically reviewed published studies on drug-resistant *M. tuberculosis* in Iran. The search terms were “*Mycobacterium tuberculosis* susceptibility” or “*Mycobacterium tuberculosis* resistant” and Iran.

**Results:** Fifty-two eligible articles, published during 1998–2014, were included in this review. Most of the studies were conducted in Tehran. The most common used laboratory method for detecting *M. tuberculosis* drug resistant was Agar proportion. The highest resistance to first-line drugs was seen in Tehran, the capital city of Iran. The average prevalence of isoniazid (INH), rifampin (RIF), streptomycin (SM), and ethambotol (EMB) resistance via Agar proportion method in Tehran was 26, 23, 22.5, and 16%, respectively. In general, resistance to INH was more common than RIF, SM, and EMB in Tehran

**Conclusions:** In conclusion, this systematic review summarized the prevalence and distribution of first-line anti-tubercular drug resistance of *M. tuberculosis* in Iran. Our results suggested that effective strategies to minimize the acquired drug resistance, to control the transmission of resistance and improve the diagnosis measures for TB control in Iran.

## Introduction

Tuberculosis (TB) remains as one of the most common infectious disease in developing countries (Nasiri et al., 2014). In 2012, ~8.6 million people developed TB and 1.3 million died from the disease (Organization, 2013). TB is an important health problem, and this issue has become even more as a result of increasing number of drug resistant strains (Shamaei et al., 2009). There is not a complete data about first-line anti-tubercular drug resistance of *Mycobacterium tuberculosis* in Iran, one of the eastern Mediterranean countries locating between Azerbaijan and Armenia and high-TB burden countries (such as Afghanistan and Pakistan). Since 1996, when the national TB control programs established in Iran, TB incidence has been declining from 34 per 100,000 to 21 per 100,000 cases in 2011(Organization, 2011). Knowledge of geographic variations is essential for monitoring of antibiotic resistance within a defined population of patients infected with *M. tuberculosis* (Bahrmand et al., 2009). Isoniazid (INH), rifampin (RIF), streptomycin (SM), and ethambotol (EMB) are first-line chemotherapeutic drugs used in TB therapy (Mohammadi et al., 2002). Resistant to at least INH and RIF, is of great concern, because it requires the use of second-line drugs that are difficult to procure and are much more toxic and expensive than the first line regimen (Merza et al., 2011). Based on national wide survey conducted in 1999, among all *M. tuberculosis* isolates tested for drug susceptibility, 10.9% were resistant to = 1 anti-TB drug, and 6.7% were resistant to both INH and RIF (Organization, 2000). It has been proved that patients infected with strains resistant to RIF will experience a higher failure rate with short-course 6 months chemotherapy (Shamaei et al., 2009). Together with delayed diagnosis and lack or inadequacy of TB control programs, the emergence of MDR *M. tuberculosis* has complicated the epidemiology of TB (Yang et al., 2011). Although a number of original articles from different regions of Iran have been published in recent years, there has not been a systematic review of these data. Therefore, the aim of this study was to summarize reports on first-line anti-tubercular drug resistance of *M. tuberculosis* in Iran.

## Materials and methods

### Literature search

“*Mycobacterium tuberculosis* susceptibility,” “*Mycobacterium tuberculosis* resistant,” “*M. tuberculosis* susceptibility,” and “*M. tuberculosis* resistant” and Iran were searched with special strategies in PubMed and Google Scholar engines. Three Persian scientific search engines “Scientific Information Database,” “IranMedex,” and “MagIran” were searched as well. Reference articles were explored. Both studies published in English and Persian were included. Gray literature and Abstracts of articles which published in congress were not explored. Search strategies were followed until 30th November 2014.

### Inclusion criteria

We sought any articles of antimicrobial susceptibility testing of *M. tuberculosis* isolates. In addition, the bibliography of each article were reviewed to identify additional relevant articles. Among English and Persian articles found with mentioned strategies, those with the following features were included in the study: (1) Full text was available. (2) An original article was performed. (3) Susceptibility data for at least one anti- tubercular drug was available. (4) The laboratory method was used.

### Exclusion criteria

Studies with at least one of the following aspects were excluded: (1) Studies that were not relevant. (2) Articles with only available abstracts (without full text). (3) Studies that did not use laboratory methods (using patients records). (4) Articles that use of second line of antimicrobial drug resistance. (5) Articles that were review. (6) Articles which contain no eligible data. (7) Case series reports. (8) Articles that sample size is too small (*N* < 10).

### Data collection

At this stage, articles with the following features were excluded as well: (1) Any articles were published both in English and Persian. (In these cases, the article published with more detailed results was chosen). (2) Duplicate publications. For all studies, we extracted the following data from the original publications. Literature identification and data extraction was performed by two researchers independently. Quality assessment of methodological sections and results of included articles was performed by use of STROBE checklist (http://www.equator-network.org).

## Results

**Figure 1:**
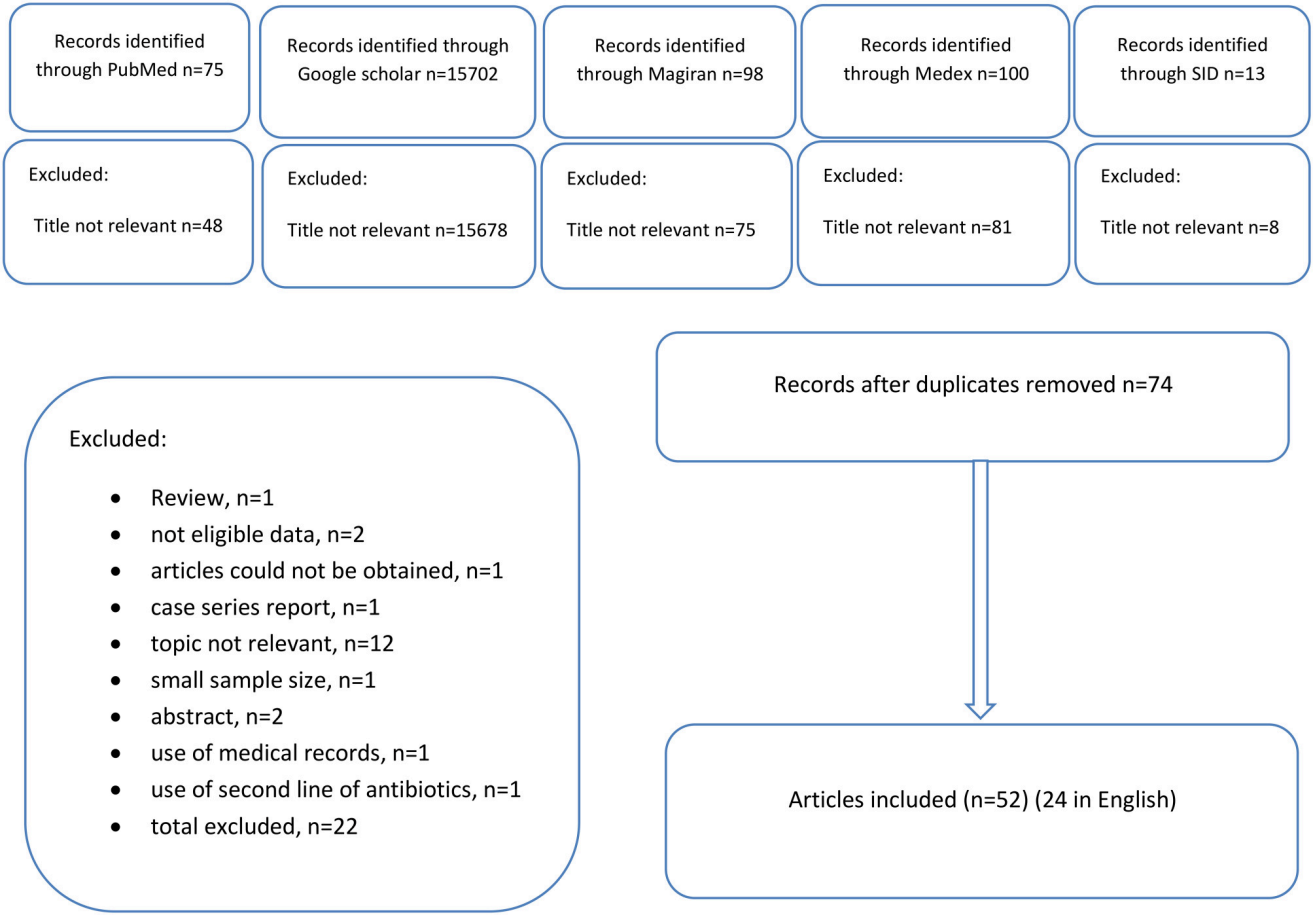
**Flow diagram of study identification**.

A total of 15,979 articles were achieved by literature search using different combination of key terms from the databases (Figure 1). After exclusion based on title not relevant and duplicates, 74 articles were retrieved for detailed full-text evaluation. Finally 52 studies, 24 in English, and 28 in Persian, addressing the prevalence of drug resistance TB were included (Tables 1, 2). The original articles were performed in different places of Iran. Most studies were conducted in Tehran (*n* = 25; Bahrmand et al., 2000; Mohammadi et al., 2002; Seyed-Davood Mansoori et al., 2003; Masjedi et al., 2006; Mirsaeidi et al., 2007; Mohammadzadeh et al., 2007; Farnia et al., 2008a,b; Shamaei et al., 2009; Dinmohammadi et al., 2010; Merza et al., 2011; Ostadzadeh et al., 2011; Sheikholslami et al., 2011; Taghavi et al., 2011; Tasbiti et al., 2011; Derakhshani Nezhad et al., 2012; Marjani et al., 2012; Mohammadi, 2012; Tahmasebi et al., 2012; Bahrami et al., 2013; Ali et al., 2014; Nasiri et al., 2014; Sheikh Ghomi et al., 2014; Varahram et al., 2014; Velayati et al., 2014) and Tabriz (*n* = 8; Hassan Heidarnejad and Nagili, 2001; Moadab and Rafi, 2006; Varshochi et al., 2006; Asgharzadeh et al., 2007, 2014; Rafi et al., 2009; Roshdi and Moadab, 2009; Zamanlou et al., 2009). Other studies were performed in Khorasan (*n* = 3; Namaei et al., 2006; Velayati et al., 2014; Sani et al., 2015), Ardebil (*n* = 1; Velayati et al., 2014), Isfahan (*n* = 3; Moniri, 2001; Nasiri et al., 2014; Velayati et al., 2014), Mazandaran (*n* = 3; Pourhajibagher et al., 2012; Babamahmoodi et al., 2014; Velayati et al., 2014), Gilan (*n* = 1; Velayati et al., 2014), Hamadan (*n* = 1; Velayati et al., 2014), Kerman(*n* = 1; Velayati et al., 2014), Kurdistan(*n* = 1; Velayati et al., 2014), Yazd (*n* = 1; Velayati et al., 2014), Qazvin (*n* = 1; Velayati et al., 2014), Kermanshah (*n* = 4; Izadi et al., 2011; Nasiri et al., 2014; Velayati et al., 2014; Mohajeri et al., 2014), Golestan (*n* = 3; Javid et al., 2009; Livani et al., 2011; Velayati et al., 2014), Markazi (*n* = 3; Farazi et al., 2013; Taherahmadi et al., 2013; Velayati et al., 2014), Lorestan (*n* = 1; Velayati et al., 2014), Khuzestan (*n* = 2; Khosravi et al., 2006; Velayati et al., 2014), Sistan va Baluchistan (*n* = 5; Bostanabad et al., 2007; Bahrmand et al., 2009; Haeili et al., 2013; Nasiri et al., 2014; Velayati et al., 2014), Qom (*n* = 1; Velayati et al., 2014), Fars (*n* = 1; Velayati et al., 2014), Hormozgan (*n* = 2; Nasiri et al., 2014; Velayati et al., 2014), and Semnan (*n* = 1; Velayati et al., 2014). A study which was conducted by Velayati et al. (2014), in years 2010–2011, has been investigated drug resistant in various places in Iran (Tehran, Sistan ba Balochestan, Khozestan, Khorasan, Ardebil, Qom, Golestan, Isfahan, Gilan, Fars, Hormozgan, Mazandaran, Semnan, Lorestan, Hamedan, Kerman, Kordestan, Kermanshah, Markazi, Yazd, and Qazvin (Velayati et al., 2014), but we identified it as 1 study in search flow diagram, it was considered for Nasiri et al. study too (Nasiri et al., 2014). In Isfahan, 4 surveys were performed but in one of them (Tavakoli et al.) only abstract was available, and it was excluded from total records. One study which was conducted by Moaddab et al. (2011) that did not note the location. One study has been done in Tehran and Zabol (Zakerbostanabad et al., 2008). One study has been conducted by Haeili et al. (2013) in Tehran, Alborz, Sistan va Blochestan, Hormozgan, and Kermanshah. Another study had been done in Tehran-Arak by Taheri et al. (2013). The reference method for determining drug resistance of *M. tuberculosis* was agar proportion. Using this method, the mean of resistance to INH, RIF, SM and EM in Iran was 20, 18, 18%, and to EM is 12%, respectively. Despite the reference method for susceptibility test is agar proportion (Rieder et al., 1998), the method that was used in most of the cities were PCR. For this reason, we determine the mean of resistance to INH and RIF in different geographical regions based on this method too. If Iran is divided into 8 geographical regions (Table 3), the mean of resistance to INH in Northern provinces of country was 5%, and maximum resistance was seen in Golestan and the minimum resistance was belonged to Gilan province. The mean of resistance to RIF was 4%, and the maximum and minimum resistance was seen in Gilan and Mazandaran, respectively. The mean of resistance to INH and RIF in Southern provinces of Iran was 6.45 and 10%, respectively. The mean of resistance to INH in Western provinces of country was 5%, and the maximum resistance belonged to Kordestan and minimum resistance was seen in Lorestan. The mean of resistance to RIF was 11%, and the highest and lowest resistance was seen in Lorestan and Kordestan. The mean of resistance to INH and RIF in Northwest provinces of Iran was 6.5 and 3%, respectively. The mean of resistance to INH in central provinces of country was 9%, and maximum resistance belonged to Markazi while minimum resistance belong to Yazd that no resistance has been seen. The mean of resistance to RIF was 10%, and the highest and lowest level of resistance was seen in Markazi and Qom. One of the provinces of central regions is Isfahan. In Isfahan the mean of resistance to INH based on agar proportion was 12.6% which was similar to PCR method (12%), but mean of resistance to RIF based on agar proportion was 26%, that was higher than PCR method (7%). In Markazi provinces, the mean of resistance to INH, based on reference method was 2.6% that was lower than PCR; there was the same result about RIF too. In Southwest of Iran, resistance to INH and RIF was 6 and 5%, respectively. The mean of resistance to INH and RIF in Northeast provinces of Iran was 4%. In Southeast provinces of Iran such as Sistan- Blochestan and Kerman, the mean of resistance to INH and RIF was 4.4 and 9% based on PCR method. Because the most of method that use in Sistan- Blochestan was agar proportion, we calculate the mean of resistance to INH and RIF based on the mentioned method, (INH 20% and RIF 12%). Due to the large number of studies in Tehran and Tabriz, these provinces were examined separately. The most common laboratory method that used was agar proportion in Tehran. The average prevalence of resistant against INH in Tehran was 26%, RIF 23%, SM 22.5%, and EMB 16% by agar proportion method. In general, resistance to INH was more common than RIF, SM and EMB in Tehran. The average prevalence of resistant against INH in Tabriz was 15%, RIF 5%, SM 19%, and EMB 2.43% by agar proportion. The highest resistance to first-line drugs was seen in Tehran. Most studies about two drug resistances were conducted in Tehran by proportional method on INH and RIF. The mean of resistance to INH and RIF in Tehran was 22% by proportional method. The mean of resistance to INH and RIF in IRAN was 15% by this method. Due to the limited number of studies on other two drug resistance, the results are given only in Table 2. Most studies on three drug resistance were conducted on INH, RIF and SM. The mean of resistance to these three drugs was 4% using proportional method. The mean of resistance to all first line drugs in IRAN was 3.57% by this method.

**Table 1:** **Summary of studies on resistance to a single drug among Mycobacterium tuberculosis isolates in Iran**.

**Study**					**Resistance to a single drug**							
**Location**	**Author**	**Years**	**Method**	**No. of isolates tested**	**INH**		**RIF**		**SM**		**EMB**	
					*N*	%	*N*	%	*N*	%	*N*	%
Ardebil	Velayati et al., 2014	2010–2011	PCR(a)	65	2	3	4	6	…	…	…	…
Fars	Velayati et al., 2014	2010–2011	PCR(a)	40	2	5	5	12.5	…	…	…	…
Gilan	Velayati et al., 2014	2010–2011	PCR(a)	39	1	2.5	2	5	…	…	…	…
Golestan	Javid et al., 2009	2008	PCR(b)	87	6	7	4	5	…	…	…	…
			Agar proportion	45	4	9	6	13	…	…	…	…
	Livani et al., 2011	…	MGIT	148	26	18	5	3	…	…	…	…
	Velayati et al., 2014	2010–2011	PCR(a)	47	3	6	2	4	…	…	…	…
Qom	Velayati et al., 2014	2010–2011	PCR(a)	61	3	5	3	5	…	…	…	…
Hormozgan	Velayati et al., 2014	2010–2011	PCR(a)	38	3	8	3	8	…	…	…	…
	Nasiri et al., 2014	2010–2012	Agar proportion	48	3	6	2	4	4	8	2	4
Hamedan	Velayati et al., 2014	2010–2011	PCR(a)	21	1	5	2	10	…	…	…	…
Isfahan	Velayati et al., 2014	2010–2011	PCR(a)	42	5	12	3	7	…	…	…	…
	Nasiri et al., 2014	2010–2012	Agar proportion	45	2	7	2	9	1	2	0	0
	Moniri, 2001	1998–2009	Agar proportion	94	17	18	41	44	14	15	3	3
Khorasan	Namaei et al., 2006	2001–2002	indirect proportion	105	1	1	…	…	27	26	…	…
	Velayati et al., 2014	2010–2011	PCR(a)	117	10	8.5	9	8	…	…	…	…
	Sani et al., 2015	2012–2013	Agar proportion	100	7	7	7	7	9	9	3	3
Kermanshah	Izadi et al., 2011	2006–2008	Agar proportion	14	8	57	6	43	…	…	…	…
	Velayati et al., 2014	2010–2011	PCR(a)	16	1	6	2	12.5	…	…	…	…
	Nasiri et al., 2014	2010–2012	Agar proportion	15	4	26.6	3	20	3	20	3	20
	Mohajeri et al., 2014	2011–2012	Agar proportion	112	18	16	16	14	25	22	15	13
Kermanshah	Mohajeri et al., 2015	2011–2013	Agar proportion	125	…	…	35	28	…	…	…	…
Khozestan	Khosravi et al., 2006	2001	PCR(c)	80	5	6	6	7.5	…	…	…	…
	Velayati et al., 2014	2010–2011	PCR(a)	119	7	6	3	2.5	…	…	…	…
Kerman	Velayati et al., 2014	2010–2011	PCR(a)	24	1	4	3	12.5	…	…	…	…
Kordestan	Velayati et al., 2014	2010–2011	PCR(a)	16	2	12.5	0	0	…	…	…	…
Lorestan	Velayati et al., 2014	2010–2011	PCR(a)	24	0	0	5	21	…	…	…	…
Mazandaran	Pourhajibagher et al., 2012	2010–2011	PCR(d)	59	4(use of katG gene) 3(use of inhA gene)	7 5	1	2	…	…	…	…
	Velayati et al., 2014	2010–2011	PCR(a)	26	1	4	1	4	…	…	…	…
	Babamahmoodi et al., 2014	…	LPA(e)	54	2	4	3	5.5	4	7	…	…
Markazi	Taherahmadi et al., 2013	…	Agar proportion PCR-RFLP (f)	60	…	…	…	…	…	…	43 19	72 32
	Farazi et al., 2013	2011–2012	Agar proportion	115	3	3	2	2	3	3	8	7
	Velayati et al., 2014	2010–2011	PCR(a)	15	3	20	3	20	…	…	…	…
Qazvin	Velayati et al., 2014	2010–2011	PCR(a)	10	1	10	0	0	…	…	…	…
Semnan	Velayati et al., 2014	2010–2011	PCR(a)	21	0	0	0	0	…	…	…	…
	Zakerbostanabad et al., 2008	2005–2006	Agar proportion	91	28	31	4	4	23	25	8	9
Sistan va Balochestan	Bahrmand et al., 2009	2005–2006	Agar proportion	286	…	…	78	27	…	…	…	…
	Velayati et al., 2014	2010–2011	PCR(a)	165	8	5	10	6	…	…	…	…
	Nasiri et al., 2014	2010–2012	Agar proportion	59	5	8	3	5	8	13.5	3	5
Tehran	Ostadzadeh et al., 2011	…	Agar proportion	50	…	…	25	50	…	…	…	…
	Farnia et al., 2008a	…	Agar proportion MGMT Both of them	60	0 0 30	0 0 50	0 0 30	0 0 50	4 3 29	7 5 48	3 5 28	5 8 47
	Sheikholslami et al., 2011	…	Agar proportion PCR-SSCP(g)	74	17 10	23 13.5	7 4	9 5	…	…	…	…
	Seyed-Davood Mansoori et al., 2003	1996–2000	Agar proportion	273	76	28	50	18.5	50	18.5	28	10
	Bahrmand et al., 2000	1998–1999	Agar proportion	563	35	6	25	4	55	10	17	3
	Mohammadi et al., 2002	1999–2000	MGIT Direct MGIT in Direct Agar proportion	15	10 10 7	67 67 47	11 11 8	73 73 53	5 6 7	33 40 47	5 5 5	33 33 33
	Dinmohammadi et al., 2010	1999–2008	Agar proportion	90	52	58	…	…	…	…	…	…
	Shamaei et al., 2009	2000–2003	Agar proportion	548	152	28	119	22	184	34	75	14
	Merza et al., 2011	2000–2005	Agar proportion	1742	414	24	307	18	478	27	207	12
	Mirsaeidi et al., 2007	2003–2004	Agar proportion	264	93	35	52	20	96	36	35	13
	Marjani et al., 2012	2003–2008	Agar proportion	554	81	15	27	5	116	21	22	4
	Varahram et al., 2014	2003–2011	Agar proportion and Allele specific PCR	4825	296	6	…	…	…	…	…	…
	Farnia et al., 2008b	2006–2007	Agar proportion	258	9	3	3	1	7	3	1	0.4
	Mohammadi, 2012	2006–2008	Agar proportion MAS-PCR(h)	90	…	…	37 29	41 32	…	…	…	…
	Tasbiti et al., 2011	2006–2009	Agar proportion	1027	116	11	110	11	232	23	104	10
	Taghavi et al., 2011	2008–2009	Agar proportion MAS-PCR(i)	96	56 43	58 45			…	…	…	…
												
	Ali et al., 2014	2009–2011	Agar proportion PCR-SSCP	103	12 5	12 5	9 4	9 4	…	…	…	…
	Velayati et al., 2014	2010–2011	PCR(a)	324	20	6	26	8				
												
	Derakhshani Nezhad et al., 2012	2010–2011	Agar proportion Allele-specific PCR	106	…	…	…	…	…	…	36 13	34 28
	Tahmasebi et al., 2012	2010–2011	Agar proportion	97	68	70	63	65	28	29	47	48
	Bahrami et al., 2013	2010–2012	Agar proportion	176	…	…	…	…	…	…	48	27
	Nasiri et al., 2014	2010–2012	Agar proportion	85	6	7	7	8	14	16	6	7
	Sheikh Ghomi et al., 2014	2012–2013	Agar proportion and Multiplex PCR	83	35	42	47	56	…	…	…	…
Tabriz	Zamanlou et al., 2009	2005–2007	Agar proportion	50	25	50	…	…	…	…	…	…
	Rafi et al., 2009	…	Agar proportion	90	6	7	3	3	17	19	…	…
	Moadab and Rafi, 2006	1999–2003	Agar proportion	90	7	8	2	2	17	19	…	…
	Asgharzadeh et al., 2007	…	Agar proportion MAS-PCR(j)	120	13	11	12	10	27	22.5	4 10	3 8
	Roshdi and Moadab, 2009	…	Agar proportion	103	2	2	0	0	8	8	0	0
	Varshochi et al., 2006	2003–2004	Agar proportion	90	20	22	9	10	28	31	5	5.5
	Hassan Heidarnejad and Nagili, 2001	…	Agar proportion	155	12	8	1	1	20	13	0	0
	Asgharzadeh et al., 2014	…	Agar proportion	120	13	11	12	10	27	22.5	4	3
Yazd	Velayati et al., 2014	2010–2011	PCR(a)	12	0	0	1	8	…	…	…	…
Tehran-Arak	Taheri et al., 2013	…	Agar proportion	40	…	…	20	50	…	…	…	…
Tehran–Alborz-Sistan va Blochestan-Hormozgan-Kermanshah	Haeili et al., 2013	2010–2012	Agar proportion	291	4	1	2	1	21	7	2	1
Tehran-Zabol-Kermanshah-Mashad-Tabriz	Bostanabad et al., 2011	2007–2008	Agar proportion	163	42	26	38	23	38	23	12	7
Unknown	Moaddab et al., 2011	…	Agar proportion and MIC	50	25	50	…	…	…	…	…	…

*(a): Multiplex-PCR assay for detection of mutations in RRDR(Rifampin resistance determinant region), and PCR assay for detection of mutations in IRDR (Isoniazid resistance determinant region) of katG, inhA*.

*(b): PCR assay for detection of mutations in RRDR of rpoB and IRDRof katG and inhA*.

*(c): PCR assay for detection of mutations in RRDR of rpoB and IRDR of katG*.

*(d): PCR assay for detection of mutations in RRDR of rpoB and IRDR of katG and inhA*.

*(e): Line Probe Assay*.

*(f): PCR-RFLP assay for detection of mutations in ERDR (Ethambotol resistance determinant region) of embB*.

*(g): PCR-SSCP assay for detection of mutations in RRDR of rpoB, ahpC and IRDR of katG, inhA*.

*(h): Mass-PCR assay for detection of mutations in RRDR of rpoB*.

*(i): Mass-PCR assay for detection of mutations in IRDR of katG*.

*(j): MAS-PCR assay for detection of mutations in ERDR of embB*.

*MGIT, Mycobacterium growth indicator tube; MIC, minimum inhibitory concentration; MGMT, malachite green microtube*.

**Table 2:** **Summary multiple drug resistance of included studies**.

**Author**	**location**	**Method**	**INH,RIF**		**INH,EMB**		**INH,SM**		**RIF,EMB**		**EMB,SM**		**RIF,SM**		**RIF,EMB,SM**		**INH,EMB,SM**		**INH,RIF,EMB**		**INH,RIF,SM**		**INH,RIF,SM,EMB**	
			***N***	**%**	***N***	**%**	***N***	**%**	***N***	**%**	***N***	**%**	***N***	**%**	***N***	**%**	***N***	**%**	***N***	**%**	***N***	**%**	***N***	**%**
Velayati et al., 2014	Ardebil	PCR	4	6																				
Velayati et al., 2014	Fars	PCR	5	12.5																				
Velayati et al., 2014	Gilan	PCR	3	8																				
Velayati et al., 2014	Golestan	PCR	2	4																				
Javid et al., 2009	Golestan	ProportionPCR	4 2	9 2																				
Livani et al., 2011	Golestan	MGIT	5	3																				
Velayati et al., 2014	Ghom	PCR	4	6.5																				
Velayati et al., 2014	Hormozgan	PCR	3	8																				
Nasiri et al., 2014	Hormozgan	Proportion	2	4																				
Velayati et al., 2014	Hamedan	PCR	0	0																				
Velayati et al., 2014	Isfahan	PCR	2	5																				
Moniri, 2001	Isfahan	Proportion	16	17			9	10	2	2			12	13							8	8.5	1	1
Nasiri et al., 2014	Isfahan	Proportion	2	4																				
Velayati et al., 2014	Khorasan	PCR	2	2																				
Namaei et al., 2006	Khorasan	Proportion									1	1					1	1						
Sani et al., 2015	Khorasan	Proportion	4	4			3	3					4	4									2	2
Velayati et al., 2014	Kermanshah	PCR	1	6																				
Izadi et al., 2011	Kermanshah	Proportion	5	36																				
Nasiri et al., 2014	Kermanshah	Proportion	3	20																				
Mohajeri et al., 2014	Kermanshah	Proportion	16	14																				
Velayati et al., 2014	Khozestan	PCR	6	5																				
Khosravi et al., 2006	Khozestan	Proportion	7	9																				
Velayati et al., 2014	Kerman	PCR	3	12.5																				
Velayati et al., 2014	Kordestan	PCR	0	0																				
Velayati et al., 2014	Lorestan	PCR	0	0																				
Velayati et al., 2014	Mazandaran	PCR	1	4																				
Babamahmoodi et al., 2014	Mazandaran	LPA	0	0																				
Velayati et al., 2014	Markazi	PCR	2	13																				
Farazi et al., 2013	Markazi	Proportion	9	8																			2	2
Velayati et al., 2014	Qazvin	PCR	2	20																				
Velayati et al., 2014	Semnan	PCR	0	0																				
Velayati et al., 2014	Sistan va Blochestan	PCR	1	1																				
Nasiri et al., 2014	Sistan va Blochestan	Proportion	3	5																				
Bahrmand et al., 2009	Sistan va Blochestan	Proportion	37	13																				
Velayati et al., 2014	Tehran	PCR	32	10																				
Tahmasebi et al., 2012	Tehran	Proportion	63	65																				
Mohammadzadeh et al., 2007	Tehran	Proportion	11	48																				
Ostadzadeh et al., 2011	Tehran	Proportion	13	26																				
Taghavi et al., 2011	Tehran	Proportion MAS-PCR	36 26	38 27																				
Masjedi et al., 2006	Tehran	Proportion																					150	12
Bahrami et al., 2013	Tehran	Proportion	10	6	12	7			19	11									8	4.5				
Shamaei et al., 2009	Tehran	Proportion	106	19																				
Mirsaeidi et al., 2007	Tehran	Proportion	43	16	0	0	23	9	0	0	2	1	4	1.5	0	0	2	1	0	0	9	3	26	10
Seyed-Davood Mansoori et al., 2003	Tehran	Proportion	42	15.5	26	9.5	40	14.5	21	7.5	22	8	23	8.5	17	6	21	7.5	21	7.5	22	8	17	6
Bahrmand et al., 2000	Tehran	Proportion	3	0.5			4	1					1	0.1					1	0.1			7	1
Farnia et al., 2008a	Tehran	MGMT	8	19																				
Nasiri et al., 2014	Tehran	Proportion	6	7																				
Sheikholslami et al., 2011	Tehran	Proportion PCR-SSCP	16 4	22 5																				
Merza et al., 2011	Tehran	Proportion	263	15																				
Marjani et al., 2012	Tehran	Proportion	12	2																				
Sheikh Ghomi et al., 2014	Tehran	Proportion and PCR	30	36																				
Imani Fooladi et al., Ali et al., 2014	Tehran	Proportion PCR-SSCP	9 3	9 3																				
Rafi et al., 2009	Tabriz	Proportion	2	2															1	1	3	3	2	2
Moadab and Rafi, 2006	Tabriz	Proportion	1	1			6	7									1	1	1	1	3	3	2	2
Asgharzadeh et al., 2007	Tabriz	Proportion	1	1			5	4									1	1			2	2	2	2
Roshdi and Moadab, 2009	Tabriz	Proportion			1	1					1	1									1	1	2	2
Varshochi et al., 2006	Tabriz	Proportion																					1	1
Hassan Heidarnejad and Nagili, 2001	Tabriz	Proportion			5	3																		
Asgharzadeh et al., 2014	Tabriz	Proportion	6	5																				
Velayati et al., 2014	Yazd	PCR	0	0																				
Haeili et al., 2013	Tehran-Alborz Sistan va Blochestan Hormozgan Kermanshah	Proportion	15	5																				

**Table 3:** **Different geographical regions of Iran**.

**Region**	**Provinces**
North	Golestan, Gilan, Mazandaran
South	Fars, Hormozgan
West	Kordestan, Kermanshah, Lorestan, Hamedan
Center	Isfahan, Qom, Markazi, Yazd
Northeast	Khorasan, Semnan
Northwest	Ardebil, Ghazvin
Southeast	Sistan-Blochestan, Kerman
Southwest	Khozestan

## Discussion

This review addressed the prevalence of first-line anti-tubercular drug resistance of *M. tuberculosis* in Iran. Various types of methods were used for determination of the susceptibility of *M. tuberclusis*: agar proportion (reference method), different types of PCR (PCR-RFLP, Real time PCR, PCR-SSCP, MAS-PCR, and Allele specific PCR), MGMT, and MGIT (direct and indirect). But most of them that used were agar proportion or PCR. In all the studies that use both of them, the results of reference method (agar proportion) had the highest of sensitivity and specificity (Javid et al., 2009; Sheikholslami et al., 2011; Derakhshani Nezhad et al., 2012; Mohammadi, 2012; Taherahmadi et al., 2013). In this study, evaluation of first-line anti-tubercular drug resistance in various provinces of Iran was based on PCR method that is not very accurate. It seems that the prevalence of drug resistance is higher than the results of studies that use mentioned method (PCR). As can be seen in the Table 1, the highest resistance of *M. tuberculosis* to first line drugs was observed in Tehran, INH:26%, RIF:23%, SM:22.5%, and EMB:16%. This could be due to transferring of patients with treatment failure to referral Hospitals in Tehran. The other reason could be presence of different nations such as Afghan, Iraq and Pakistan in Tehran. Between 1996 to 2000, three studies have been conducted in Tehran, Mohammadi et al. (2002), Bahrmand et al. (2000), and Seyed-Davood Mansoori et al. (2003) reported the resistance prevalence of 46.6, 6.2, and 28% to isoniazid, respectively. The reason of this difference could be due to small sample size in first study (Mohammadi et al., 2002). In Mohamadi et al. study, *M. tuberculosis* was isolated from referral patients that can be the reason of high resistance to isoniazid in this study. Two studies have been conducted in 2010–2011, in Velayati et al. (2014) reports, the prevalence of isoniazid resistance was 6% and in Tahmasebi et al. (2012) this level was 70.1%, that the reason of this difference could be used to strains that isolate from patients with treatment failure in second study. Over the years the increasing level of resistance to isoniazid might be due to incomplete treatment. The failure treatment can be for two reason, inappropriate drug prescribing and drug usage regularly and on time. This process has been seen about rifampin resistance. During 2000 and 2008, Shamaei et al. (2009) and Merza et al. (2011) reported the highest prevalence of rifampin resistance. These studies have been done in Masih daneshvari Hospital that is a referral hospital in Iran and most of the patients refer to this hospital due to treatment failure. As mentioned in results, high prevalence of resistance to INH (20%) and RIF (12%) was seen in Sistan va Blochestan due to vicinity of this province to Afghanistan and Pakistan. Rifampin and isoniazid resistance is a surrogate marker for MDR- *M. tuberculosis*. Most of studies reporting isoniazid and rifampin resistance were conducted in Tehran. These studies report the highest prevalence of resistance to INH and RIF (22%). In Masjedi et al. (2008) study, among 77% Iranian and 23% afghan cases, 131 Iranian (65%), and 13 afghan cases (22%) were susceptible to all 4 drugs tested and 72 patients (28%) were MDR-TB case. Notably, 38 MDR-TB cases (52.7%) were isolated from afghan immigrants. Twenty patients (47%) had mono drug resistant strains (nine were INH, seven SM, three RF, and one EMB mono resistant) and 22 (52%) had combined resistance.

In Al-Akhali et al. (2007) study that was performed in Yemen, the prevalence of resistance to any one of the four drugs was 9.8% in the new cases and 17.4% in the previously treated cases. The prevalence of MDR-TB, defined as TB cases excreting *M. tuberculosis* resistant at least to INH and RIF, was 3%. In Ayaz et al. (2012) study that conducted in Pakistan, resistance to one or more of the first-line anti-TB drugs was noted in 23% of patients. The INH resistance was 9% in untreated and 28.5% in treated patients. Resistance to other first-line drugs was as follow: SM 17%, EMB 5%, and RIF 5%.

Some limitations of this systematic review should be considered for results interpretation. First, few studies have been conducted in our country about resistance of TB to first and second line-drugs. Second, the probable influence of age, sex, ethnicity, economic level, and life styles could not be analyzed due to the limited information obtained from the original articles. Third, most included studies were hospital-based rather than population based which makes the results more prone to potential selection bias. Because of the small number of studies particularly in other cities except Tehran, we cannot judge about the prevalence of resistance against first-line anti-tuberculosis drugs properly. However, in recent years, emergence and spread of MDR-TB threaten the TB control strategy. In many law-and middle-income countries, due to inadequate laboratory capacity, most of the patients with MDR-TB are not diagnosed. Treatment of these cases mostly failed and significant expenditure of health care resources is needed.

In conclusion, this systematic review summarized the prevalence and distribution of first-line anti-tubercular drug resistance of *M. tuberculosis* in Iran. Our results suggest effective strategies to minimize the acquired drug resistance, to control the transmission of resistance and improve the diagnosis measures for TB control in our country.

An important element in gaining control of this epidemic is developing an understanding of the molecular basis of resistance to the most important anti-tuberculosis drugs. Since the mechanism of action of rifampin is to inhibit mycobacterial transcription by targeting DNA-dependent RNA polymerase (Somoskovi et al., 2001), routine application of rapid molecular tests in the clinical management of drug-resistant tuberculosis is highly recommended.

On the other hand, INH is activated by the mycobacterial enzyme KatG, a multifunctional catalase-peroxidase that has other activities including peroxynitritase and NADH oxidase. Therefore, inhibition of both cell wall lipid, and nucleic acid synthesis by INH-NAD and INH-NAPD adducts together with respiratory inhibition by INH-derived NO can provides a potent antituberculosis cocktail. Some strategies such as developing agents that produce the isonicotinoyl radical, screening for molecules which increase mycobacterial levels of NAD+ or NADP+ for in co-administration use with INH, to designing of more drug-like molecules using the structure of INH-NAD adducts to inhibit specifically mycobacterial enzymes; and developing of mycobacterial enzyme inhibitors which can inactivate INH might be useful to control INH-TB resistance propagation (Timmins and Deretic, 2006).

## Author contributions

All authors listed, have made substantial, direct and intellectual contribution to the work, and approved it for publication.

### Conflict of interest statement

The authors declare that the research was conducted in the absence of any commercial or financial relationships that could be construed as a potential conflict of interest.
